# The Hippo signaling pathway contributes to the 2,5-Hexadion-induced apoptosis of ovarian granulosa cells

**DOI:** 10.1186/s13048-023-01249-4

**Published:** 2023-08-11

**Authors:** Yi Sun, Huiting Chen, Sichuan Chen, Xueming Xu, Wenchang Zhang, Yuchen Li

**Affiliations:** 1 Department of Preventive Medicine, Fujian Provincial Key Laboratory of Environmental Factors and Cancer, Key Laboratory of Environment and Health, School of Public Health, Fujian Medical University, Fuzhou, 350122 Fujian Province China; 2 Key Laboratory of Environment and Female Reproductive Health, The Eighth Affiliated Hospital, Sun Yat-sen University, Shenzhen, China

**Keywords:** 2,5-hexanedione, Ovarian granulosa cells, Hippo signaling pathway, Apoptosis, mRNA expression microarray

## Abstract

Although n-hexane can induce ovarian damage by inducing ovarian granulosa cell (GC) apoptosis, the mechanism underlying this induction of apoptosis has not been fully elucidated. In this study, rat ovarian GCs were exposed to different concentrations of 2,5-hexanedione (2,5-HD) (the main metabolite of n-hexane) in vitro to observe apoptosis, and the mechanism was further explored via mRNA microarray analysis. Hoechst 33258 staining and flow cytometry suggested that the apoptosis rate of ovarian GC apoptosis was significantly increased in the 2,5-HD-treated group. Subsequently, microarray analysis revealed that a total of 5677 mRNAs were differentially expressed, and further GO and KEGG analyses revealed that the differentially expressed genes were significantly enriched in many signaling pathways, including the Hippo pathway. A total of 7 differentially expressed genes that function upstream of the Hippo signaling pathway (Nf2, Wwc1, Ajuba, Llgl1, Dlg3, Rassf6 and Rassf1) were selected to confirm the microarray results by qRT-PCR, and the expression of these genes did change. Subsequently, the expression of key effector genes (Yap1, Mst1 and Lats1) and target genes (Ctgf and Puma) of the Hippo signaling was measured, and the results suggested that the mRNA and protein levels of Yap1, Mst1, Lats1, and Ctgf were significantly decreased while those of Puma were significantly increased after 2,5-HD treatment. Further CO-IP analysis suggested that the interaction between YAP1 and TEAD was significantly reduced after 2,5-HD treatment, while the interaction between YAP1 and P73 was not affected. In summary, during the 2,5-HD-induced apoptosis of ovarian GCs, the Hippo signaling pathway is inhibited, and downregulation of the pro-proliferation gene Ctgf and upregulated of the pro-apoptosis gene Puma are important. Decreased Ctgf expression was associated with decreased binding of YAP1 to TEAD. However, increased PUMA expression was not associated with YAP1 binding to P73.

**Supplementary Information:**

The online version contains supplementary material available at 10.1186/s13048-023-01249-4.

## Introduction

2,5-Hexadione (2,5-HD) is the main metabolite of n-hexane, and it is considered a substance that plays a key role in the toxic effects of n-hexane [17]. N-Hexane is an organic solvent that is commonly used in industry, including the footwear and leather industries [28]. Occupational exposure to n-hexane during production causes frequent chronic n-hexane poisoning events, resulting in toxicity in multiple organs and systems, including the reproductive and nervous systems [17, 28]. In recent years, the toxic effects of n-hexane on reproductive systems have received increasing attention. In particular, n-hexane causes female reproductive toxicity. Limited studies have shown that n-hexane and 2,5-HD can affect the normal growth and development of ovarian follicles and interfere with the secretion of sex hormones [1, 18]. Oral exposure to 2,5-HD increases follicle-stimulating hormone and progesterone levels and decreases estradiol levels in rats [1]. However, the mechanism underlying the toxic effects of 2,5-HD on the female reproductive system has not yet been fully elucidated.

Ovarian granulosa cells (GCs) are the main cell type in the ovary, and these cells participate in steroid hormone synthesis and the maintenance of normal follicular development. The proliferation and apoptosis of GCs affect follicular development and ovulation [10], and GC apoptosis is the direct cause of follicular atresia [22]. Zhang et al showed that treatment of primary rat ovarian GCs with 2,5-HD decreased cell viability and increased apoptosis [41]. In addition, exposure of human ovarian GCs to 2,5-HD for 24 h increased apoptosis by decreasing BCl2 expression and increasing Bax expression [30]. Ovarian GC apoptosis may be one of the main mechanisms underlying the toxic effects of n-hexane and its main metabolite 2,5-HD on ovaries.

Although domestic and foreign scholars have discussed the mechanism underlying n-hexane- and 2,5-HD-induced ovarian GC apoptosis, knowledge about this topic is still relatively limited. The Hippo signaling pathway is a signal transduction pathway that was discovered and has been widely studied in recent years; this pathway functions to maintain the balance between cell proliferation and apoptosis and the stability of the intracellular environment [6, 15]. When the Hippo pathway is inhibited, activated YAP translocates to the nucleus and promotes the expression of genes that regulate cell proliferation and apoptosis [36]. YAP is a downstream effector molecule of the Hippo pathway and can be involved in the regulation of ovarian function [2]. It has been suggested that the Hippo pathway can participate in primordial follicle initiation by regulating AKT signaling molecules in mice [14], and YAP knockdown in human ovarian GCs results in a significant decrease in cell proliferation [8]. Thus, the Hippo signaling pathway may play an important role in the regulation of ovarian GC apoptosis.

In this study, we aimed to preliminarily explore genes that are differentially expressed during 2,5-HD-induced ovarian GC apoptosis by mRNA expression profiling technology and to investigate select signaling pathways (such as the Hippo signaling pathway) that may be closely related to ovarian GC apoptosis. Furthermore, qRT-PCR, western blot and Co-IP were used to measure the expression levels of genes and proteins that are related to the Hippo signaling pathway. We also aimed to analyze the interactions of the key signaling molecule YAP with transcription factors that are related to cell proliferation and apoptosis after its nuclear translocation in order to investigate the possible molecular mechanism by which the Hippo signaling pathway regulates 2,5-HD-induced ovarian GC apoptosis. We hope to provide new insights into female reproductive toxicity studies of 2,5-HD.

## Materials and methods

### Animals

Twenty-one-day-old female SD rats of clean grade (50~60 g) were purchased from the Laboratory Animal Center of Fujian Medical University (production license number: SCXK 2018-0002). The rats were fed and given water ad libitum, and the environmental conditions of the room were controlled by a central air conditioner and maintained at a temperature of 20~26 ℃ and a relative humidity of 50%~70%. After one week of adaptive feeding, bilateral ovaries were aseptically harvested from the backs of the rats (48 h before the ovaries were harvested, the rats were injected with pregnant mare serum gonadotropin (PMSG, 10 IU, Ningbo Company, China) to stimulate follicular development). Both ovaries were transferred to culture dishes containing prewarmed dissection medium. In brief, after the back skin of the rats was disinfected with 75% alcohol, the back skin of the rats was grasped using a pair of fine dissection forceps, and a large incision was made in the skin and body wall. Next, the muscle was pushed aside using watchmaker forceps, and the ovaries were exposed. The ovaries were gently grasped with watchmaker forceps, and scissors were used to sever their attachment to the uterus. These methods were described in detail in our previous study [20].

### Isolation, culture and 2,5-HD treatment of ovarian GCs

After both ovaries were removed from the rats under aseptic conditions, residual connective tissue and blood were removed, placed in a transfer dish containing DMEM/F12 (Invitrogen, CA, USA) culture medium (supplemented with 100 U/ml penicillin G and 100 mg/L streptomycin), and incubated at 37 ℃. Follicles were punctured with a 5-gauge needle under a stereomicroscope to release GCs into the culture medium. All the media containing cells were collected, and the samples were centrifuged at 1000 rcf at 4 °C for 5 min. The supernatants were discarded, DMEM/F12 medium supplemented with 10% BSA and double antibodies was added to the cell pellets at the bottom of the centrifuge tubes to generate single cell suspension. The cell suspensions were gently mixed and transferred to culture dishes. Than, after 24 h of culture in a 37 °C cell incubator, the culture medium was replaced, and the cells were exposed to four concentrations of 2,5-HD, namely, 0 mM, 20 mM, 40 mM, and 60 mM for 24 h for subsequent experiments. The procedure was performed as previously described [31, 41].

The doses were selected based on the following. Significant toxic effects on cellular organelles and processes have been observed in both in vivo and in vitro studies when 2,5-HD was used at doses in the millimolar range [11, 21]. It was observed that 24 h of exposure to 2,5-HD (20-60 mM) can induce the apoptosis of rat pheochromocytoma (PC12) cells [29]. After 48 h of treatment with 10-30 mM 2,5-HD, the viability of human neuroblastoma SK-N-SH cells sharply decreased, with an IC_50_ of 22.4±0.2 mM, and there was a significant increase in apoptotic nucleoid formation after treatment with 8.5-17 mM 2,5-HD [39]. Therefore, in this study, ovarian GCs were treated with a relatively high dose of 2,5-HD. In addition, we performed a CCK8 assay after ovarian GCs were treated with 2,5-HD, and the results indicated that the cell viability was high (> 80%) after treatment with 0, 20, 40 and 60 mM 2,5-HD.

### Cell Counting Kit-8 (CCK-8) assay

The CCK-8 method (Dojindo Laboratories, Japan) was used to assess the relative viability of ovarian GCs. GCs were seeded in 96-well plates at a density of 1 x 10^4^ cells/well. After 24 h of treatment with 2,5-HD, 10 μL CCK-8 solution was added to each well and incubated in a 37 °C incubator (ESCO, Singapore) for 2 h. Then, the absorbance values were measured at a wavelength of 450 nm using an enzyme-labeled instrument. Cell viability was calculated with the following formula: (OD value - blank)/(OD value - blank) × 100%.

### Hoechst 33258 fluorescence staining

Apoptosis was analyzed with a Hoechst 33258 kit. Briefly, clean coverslips were placed in six-well plates, and cells were seeded in the wells at a density of approximately 50% to 80% and cultured overnight. 2,5-HD (0, 20, 40 and 60 mM) was added and incubated for 24 h. The cells were washed with PBS and incubated in 4% paraformaldehyde solution for 30 min at room temperature. The cells were then washed twice with Buffer A of the Hoechst 33258 staining kit, stained with Hoechst 33258 for 10 min, and imaged under a fluorescence microscope (100× magnification). Four quadrants of the coverslip and three fields were randomly photographed, and a total of 12 fields were imaged and analyzed. Cells with dark blue fluorescent spots in the nucleus were considered apoptotic cells. According to the difference in the fluorescence of apoptotic cells, the rate of GC apoptosis in each treatment group was calculated.

### Flow cytometry assay

GCs (*n* = 3/group) were collected via trypsin digestion without EDTA and analyzed using an apoptosis detection kit (Jiangsu Key GEN Biological Technology Development Co., Ltd., Nanjing, Jiangsu, China). Briefly, approximately 1-5×10^5^ exposed ovarian GCs were transferred to EP tubes and washed with PBS, and 100 μl of Binding Buffer was added to resuspend the cells. Then, the cells were stained with 5 μl Annexin V-FITC for 5 min, followed by 5 μl PI at room temperature in the dark for 5-15 min. Approximately 400 μL Binding Buffer was added, and flow cytometry was performed within 1 h (BD Biosciences, CA, USA).

### Microarray analysis

Ovarian GCs (*n* = 3/group) exposed to 0 mM or 60 mM 2,5-HD for 24 h were collected for mRNA microarray analysis, which was performed by Beijing Boao Jingdian Biotechnology Co., Ltd. Specifically, total RNA was extracted using the TRIzol method, and RNA samples that passed quality inspection were further purified with a mirVana™ miRNA Isolation Kit (AM1561). Subsequently, mRNA expression profiling was performed by in vitro amplification and fluorescence labeling starting with the total RNA of the test samples, and the labeling process was performed using the Crystal Core ® Biochip Universal Labeling Kit. The labeled products were purified and quantified for chip hybridization. At the end of hybridization, the chips were removed and washed in a Boo Slide Washer8 chip washer, and the washed chips were scanned using an Agilent chip scanner (G2565CA) to obtain hybridization images. Finally, the hybridization images were analyzed using Agilent Feature Instrument (v10.7) software, and data were normalized by Agilent GeneSpring software.

### Gene Ontology (GO) and Kyoto Encyclopedia of Genes and Genomes (KEGG) analyses

mRNAs that were differentially expressed (fold change (FC) ≥ 2.0 and *P* ≤ 0.05) were screened for GO and KEGG analyses. GO analysis can be divided into three parts: biological process (BP), cellular component (CC) and molecular function (MF). According to the corresponding ID or sequence annotation, relevant proteins or genes can be matched to their corresponding GO numbers and terms, that is, corresponding functional categories or cellular localization. According to the function of molecular involvement, KEGG pathway analysis was performed by analyzing the degree of target gene enrichment in signaling pathways to identify significantly enriched biological signaling pathways, and these analysis were achieved by DAVID.

### Reverse transcription and real-time quantitative PCR

TRIzol reagent (Ambion, US) was used to extract total RNA from GCs (*n* = 3/group). The PrimeScript ™ RT kit and Mir-XmiRNA First-Strand Synthesis kit (Takara, Biotechnology, Dalian, China) were then used to reverse transcribe RNA into cDNA. Finally, qRT-PCR was performed using TB GreenTM Premix EX TaqTM RR420 (Takara Bio Inc., Shiga, Japan), and gapdh was used as an internal reference. Amplification was performed on a LightCycler 480 Real-Time PCR System (Roche, Switzerland). The primer sequences are listed in Table S1. This experimental protocol was described in our previous study [19].

### Western blotting

Total protein was extracted from GCs (*n* = 3/group), and the protein concentration was determined with a BCA protein assay kit (Beyotime, Jiangsu, China). Subsequently, protein samples were added to a gel for SDS‒PAGE, and at the end of electrophoresis, the proteins were transferred to polyvinyldifluoride (PVDF) membranes. The membranes were then incubated with primary antibodies (antibodies against p-YAP1, p-MEST1, YAP1, MEST1, p-LAST1, and PUMA, 1: 1000, Cell Signaling Technology; antibodies against LAST1 and CTGF, 1:1000, Abcam) at 4 °C overnight. The membranes were washed using TBS-T and then incubated with a secondary antibody (goat anti-rabbit IgG(H+L), horseradish peroxidase (HRP) conjugate, 1:3000, Proteintech) at room temperature for 1 h. Finally, the membranes were analyzed by enhanced chemiluminescence.

### Co-IP

Cells were washed twice with PBS, and an appropriate volume of cell lysis buffer, supplemented with protease inhibitors (100×) was added according to the cell number, and the cells were thoroughly lysed on ice for 20 to 30 min. Then, 1/10 volume was collected and used as the input sample; 6/10 volume was collected and used as the IP group; and 3/10 volume was collected and used as the IgG group. Approximately two to five micrograms of antibody was added to the IP group, and 1 ul of IgG was added to the IgG group; then, the samples were incubated at 4 °C overnight with shaking in a vertical mixer. Protein-A/G bead suspensions were prepared, and 0.2 m1 Protein A/G-MagBeads were added to the IP and IgG groups and incubated at 4 °C with shaking in a vertical mixer for 1 to 2 h. After the beads were washed with Wash Buffer I, elution buffer was added, the proteins were denatured and eluted in a boiling water bath for 10 min, and the supernatant was collected. One-fourth volume of 5 × SDS loading buffer was added to each of the input, IP, and IgG samples, and the samples were boiled in a water bath for 5 min. The protein complexes were analyzed by western blotting analysis (antibody information: anti-p73 antibody, Abcam; anti-YAP antibody, TEAD Cell Signaling Technology).

### Statistical analysis

Statistical analysis was performed using SPSS 24.0, and data are presented as the mean ± standard deviation. Comparisons between two samples were performed using a t-test. Quantitative data conformed to a normal distribution, and variance was homogeneous. One-way analysis of variance (one-way ANOVA) was performed, and pairwise comparisons were performed with LSD. Dunnett’s T3 test was used for pairwise comparisons if the variance was heterogeneous, and nonnormal data were analyzed by a nonparametric rank sum test. The test level α was 0.05, and *P* < 0.05 was considered to indicate statistically significant differences.

## Results

### Morphological changes in GCs after 2,5-HD treatment

**Fig. 1:**
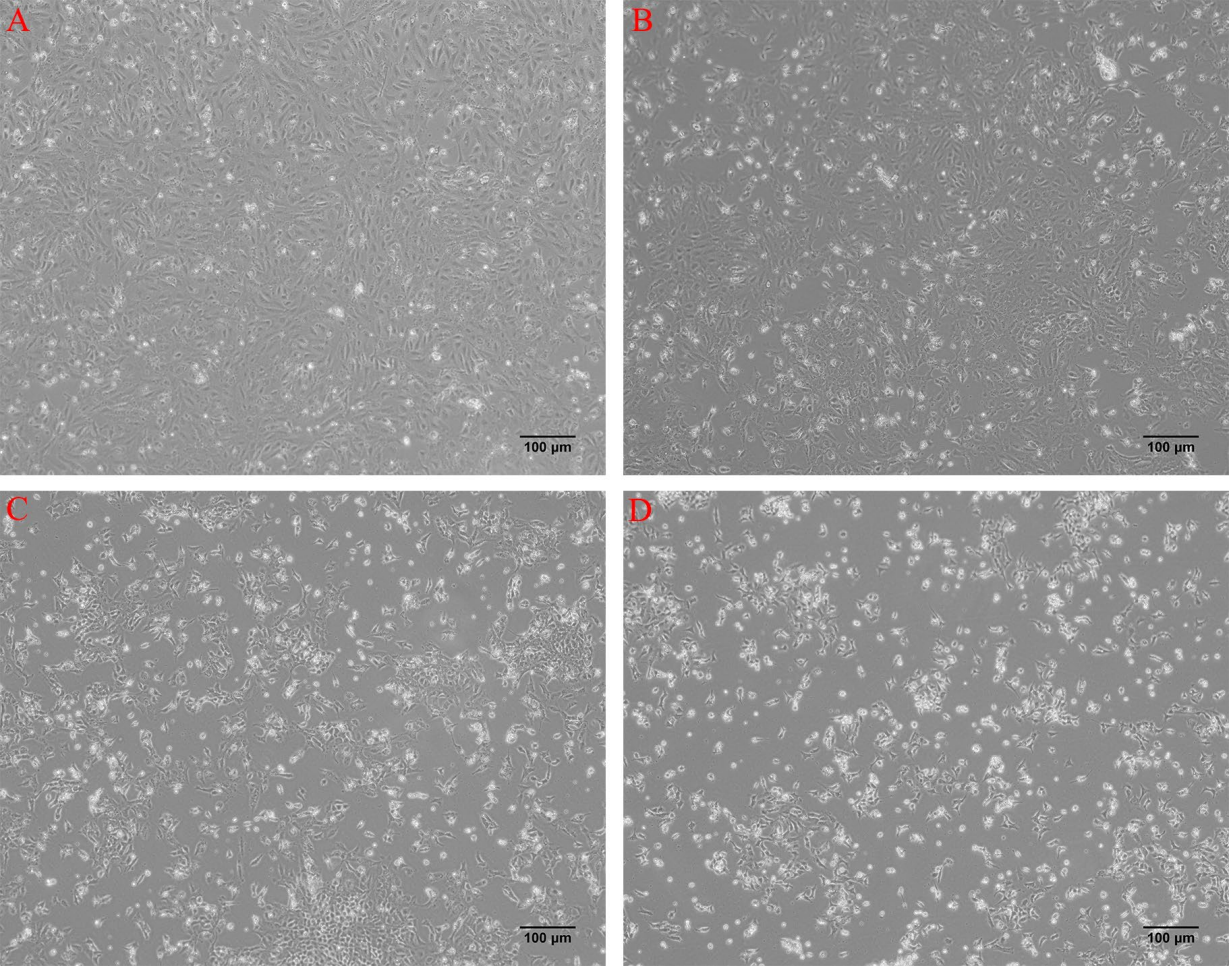
2,5-HD treatment of GCs (× 100). **A **0 mM, **B **20 mM, **C **40 mM, **D **60 mM

As shown in Fig. 1, according to microscopy, the GCs in the control group were spindle-shaped and uniform in size, with more adherent cells and tight cell-cell contact (Fig. 1A); the morphology of the GCs in the 20 mM group began to change, some GCs lost their spindle shape, and cell attachment decreased (Fig. 1B); the GCs in 40 mM group began to change from adherent long spindles to round cells, and the proportion of irregularly shaped cells gradually increased (Fig. 1C); and the GCs in the 60 mM group exhibited irregular morphology, weakened refraction, loose cell contact, and even cell membrane rupture (Fig. 1D). After 24 h of 2,5-HD treatment, the CCK8 results indicated that the cell viability was still high (>80%) in each treatment group (Figure S1).

### Apoptosis of ovarian GCs treated with 2,5-HD

**Fig. 2:**
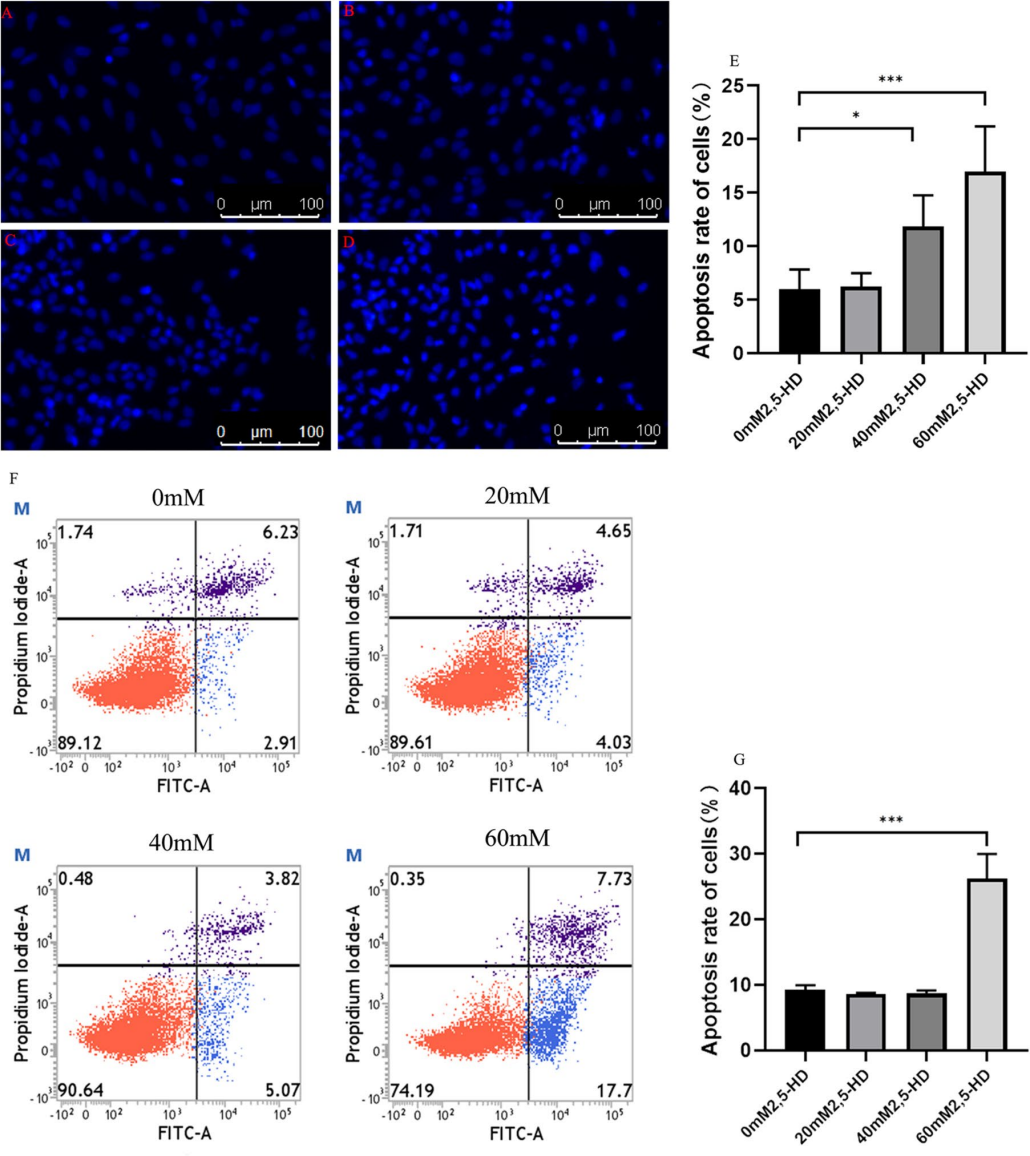
Levels of apoptosis in ovarian GCs after 2,5-HD treatment. **A **0 mM, **B **20 mM, **C **40 mM, **D **60 mM, **E** Hoechst staining was used to detect the apoptosis of ovarian GCs after 2,5-HD treatment, **F**, **G **Flow cytometry was used to measure the rate of ovarian GC after 2,5-HD treatment

First, we measured the degree of ovarian GC apoptosis by two methods. The Hoechst fluorescence staining results suggested that after ovarian GCs were treated with 2,5-HD for 24 h, the number of nuclei with strong blue fluorescence was significantly increased, and the rate of GC apoptosis was significantly higher in the 40 mM and 60 mM 2,5-HD treatment groups than in the control group (*P* < 0.05) (Fig. 2A-E). In addition, the flow cytometry results suggested that the rate of cell apoptosis in the 60 mM 2, 5-HD group was significantly higher than that in the control group (*P* < 0.05) (Fig. 2F, G).

### mRNA microarray and bioinformatics analysis

To further investigate the possible mechanism underlying 2,5-HD-induced ovarian GC apoptosis, mRNA microarray analysis was used to discover possible differentially expressed genes. Figure S2 shows the clustering plot, scatter plot and volcano plot of the microarray analysis results, and the microarray screening results suggested that the expression of 5677 mRNA molecules was changed compared with the control group (FC ≥ 2,* P* ≤ 0.05); among these molecules, 2982 genes were upregulated and 2695 genes were downregulated. Gene expression was significantly affected by 2,5-HD exposure.

**Fig. 3:**
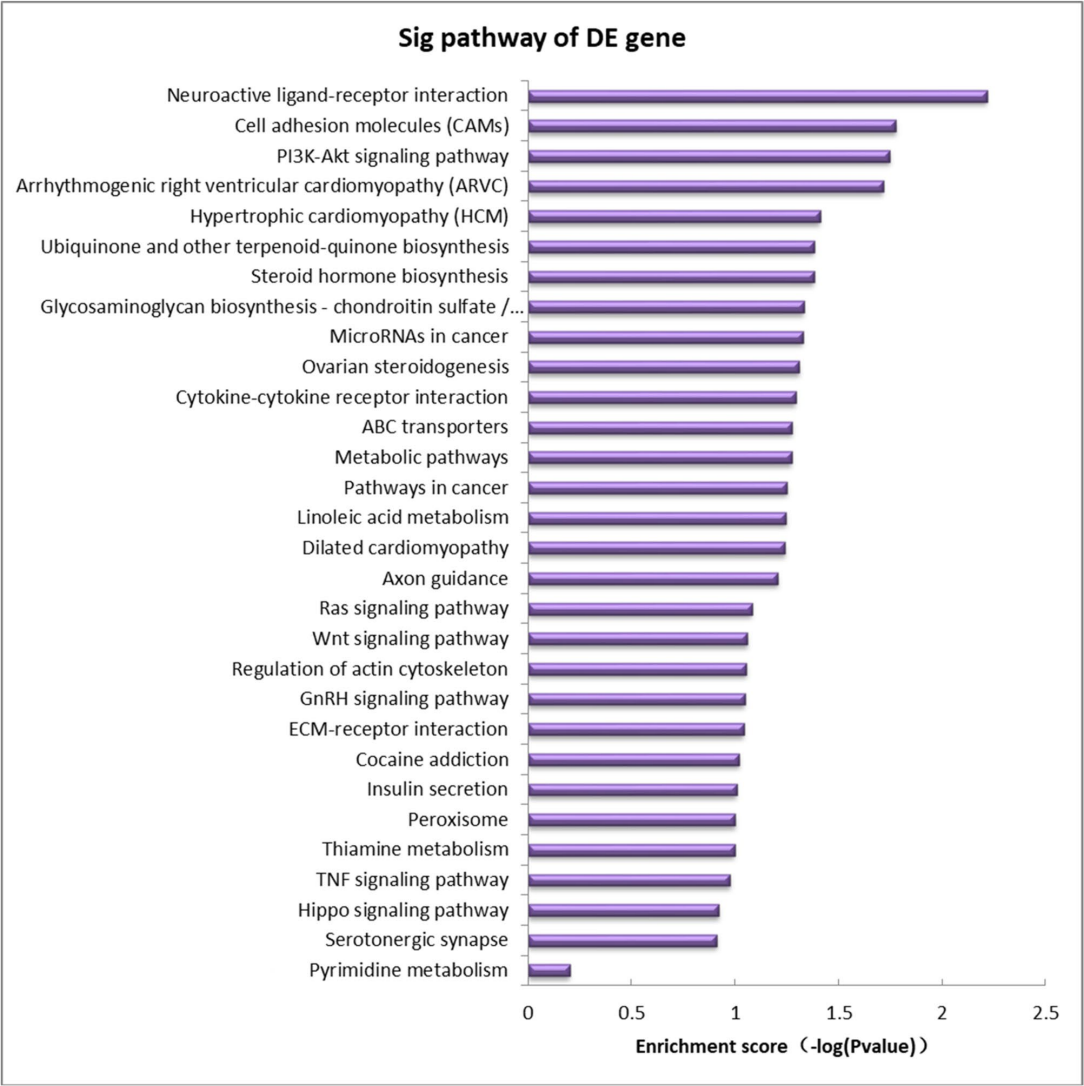
Differentially expressed gene pathway analysis (Top30, *P* < 0.05)

Subsequently, we performed GO and KEGG analysis of the differentially expressed genes, and the GO analysis results suggested that the differentially expressed genes were significantly enriched in items such as system development, developmental process, cell differentiation, and signaling activity (Table 1). In addition, the KEGG results indicated that the differentially expressed genes were significantly enriched in the Ras signaling pathway, PI3K-Akt signaling pathway and Hippo signaling pathway. This finding attracted our attention due to the close relationship between the Hippo signaling pathway and proliferation and apoptotic processes (Fig. 3).

**Table 1:** GO analysis results of differentially expressed mRNAs

**GO.ID**	**Term**	**Enrichment score**
**Biological Process**		
GO:0007275	multicellular organismal development	6.321654736
GO:0044767	single-organism developmental process	5.041611829
GO:0048731	system development	4.777046162
GO:0032502	developmental process	4.768734607
GO:0048856	anatomical structure development	4.453664786
GO:0007399	nervous system development	3.923618827
GO:0007423	sensory organ development	3.754145953
GO:0030154	cell differentiation	3.602051827
GO:0061061	muscle structure development	3.461222487
GO:0043583	ear development	3.449166625
...	...	…
**Molecular Function**		
GO:0005102	receptor binding	3.629180403
GO:0004222	metalloendopeptidase activity	2.720560299
GO:1901338	catecholamine binding	2.585346644
GO:0005249	voltage-gated potassium channel activity	2.519359047
GO:0008373	sialyltransferase activity	2.311249421
GO:0015368	calcium:cation antiporter activity	2.264286554
GO:0035250	UDP-galactosyltransferase activity	2.254803061
GO:0008009	chemokine activity	2.116991007
GO:0004769	steroid delta-isomerase activity	1.956736383
GO:0042605	sphingolipid binding	1.492154783
...	…	...
**Cellular Component**		
GO:0043197	dendritic spine	2.874983532
GO:0044309	neuron spine	2.724720441
GO:0005641	nuclear envelope lumen	2.264286554
GO:0042612	MHC class I protein complex	2.228621192
GO:0032589	neuron projection membrane	2.019749583
GO:0032777	Piccolo NuA4 histone acetyltransferase complex	1.956736383
GO:0034705	potassium channel complex	1.91762543
GO:0097458	neuron part	1.894100317
GO:0043198	dendritic shaft	1.676560399
GO:0071546	pi-body	1.602565457
...	…	…

### Measurement of Hippo signaling pathway-related mRNA expression levels by microarray

**Fig. 4:**
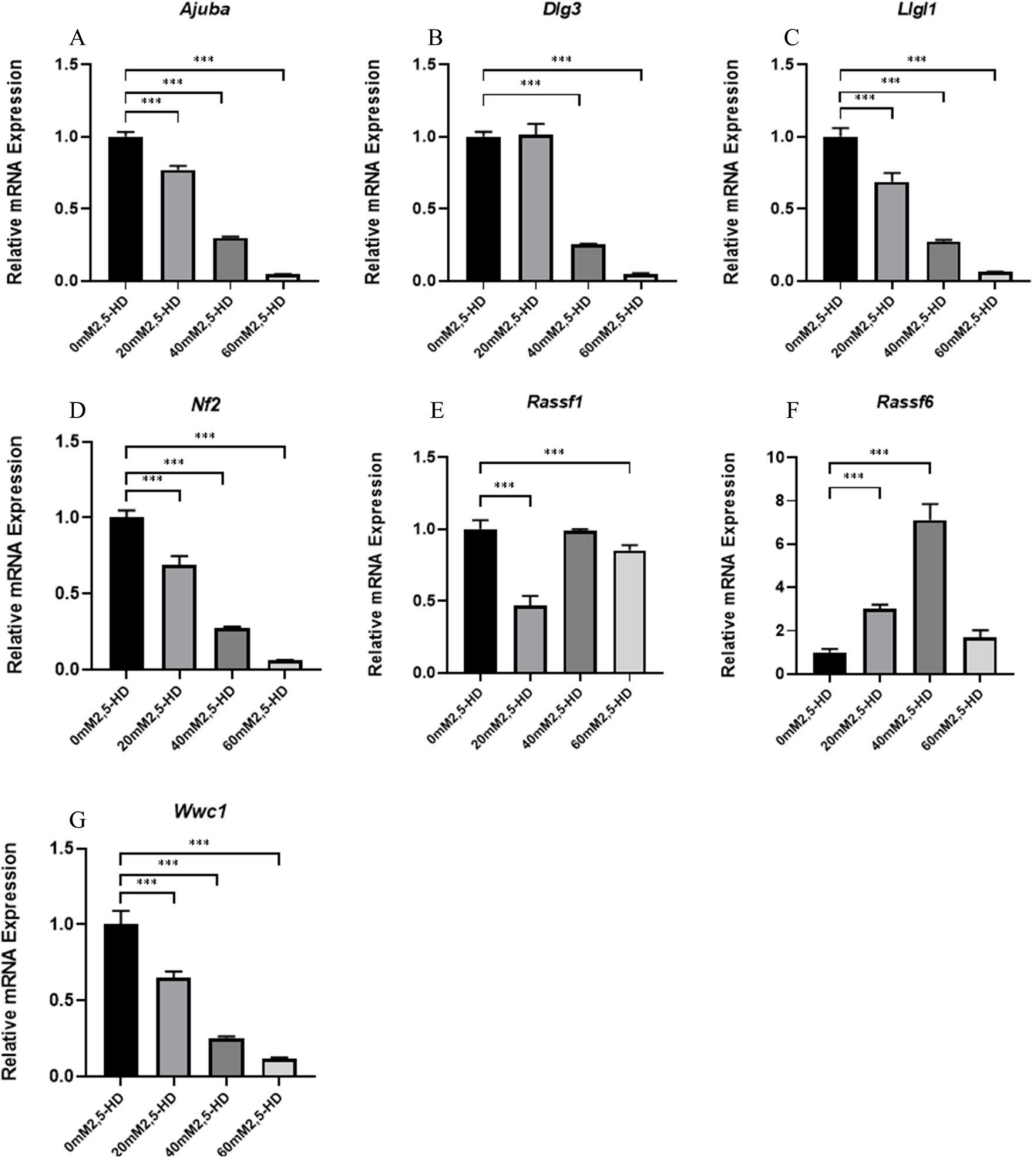
Microarray differential Hippo pathway-related mRNA expression levels after 5-HD treatment

To further elucidate whether the Hippo signaling pathway is involved in 2,5-HD-induced ovarian GC apoptosis, we screened a total of seven differentially expressed genes that are related to the Hippo signaling pathway (Nf2, Wwc1, Ajuba, Llgl1, Dlg3, Rassf6, and Rassf1) via microarrays and measured their mRNA levels. As shown in Fig. 4, compared with the control group, the expression levels of Nf2, Wwc1, Ajuba and Llgl1 were decreased in each treatment group; the expression levels of Dlg3 were decreased in the 40 mM and 60 mM treatment groups; the expression levels of Rassf6 and Puma were increased in the 20 mM and 40 mM treatment groups; and the expression levels of Rassf1 were decreased in the 20 mM and 60 mM treatment groups (*P* < 0.05). As shown in Table 2, the qRT-PCR validation results were further compared with the microarray results, and the expression results were highly consistent. The Hippo signaling pathway may indeed be altered during the 2,5HD-induced apoptosis of ovarian GCs

**Table 2:** Microarray results compared to qRT-PCR results

Gene Name	RT-PCR	Microarray
*Nf2*	down	down
*Wwc1*	down	down
*Ajuba*	down	down
*Llgl1*	down	down
*Dlg3*	down	down
*Rassf6*	up	up
*Rassf1*	up	down

### Gene expression levels of major Hippo pathway components during 2,5-HD-induced apoptosis of ovarian GCs

**Fig. 5:**
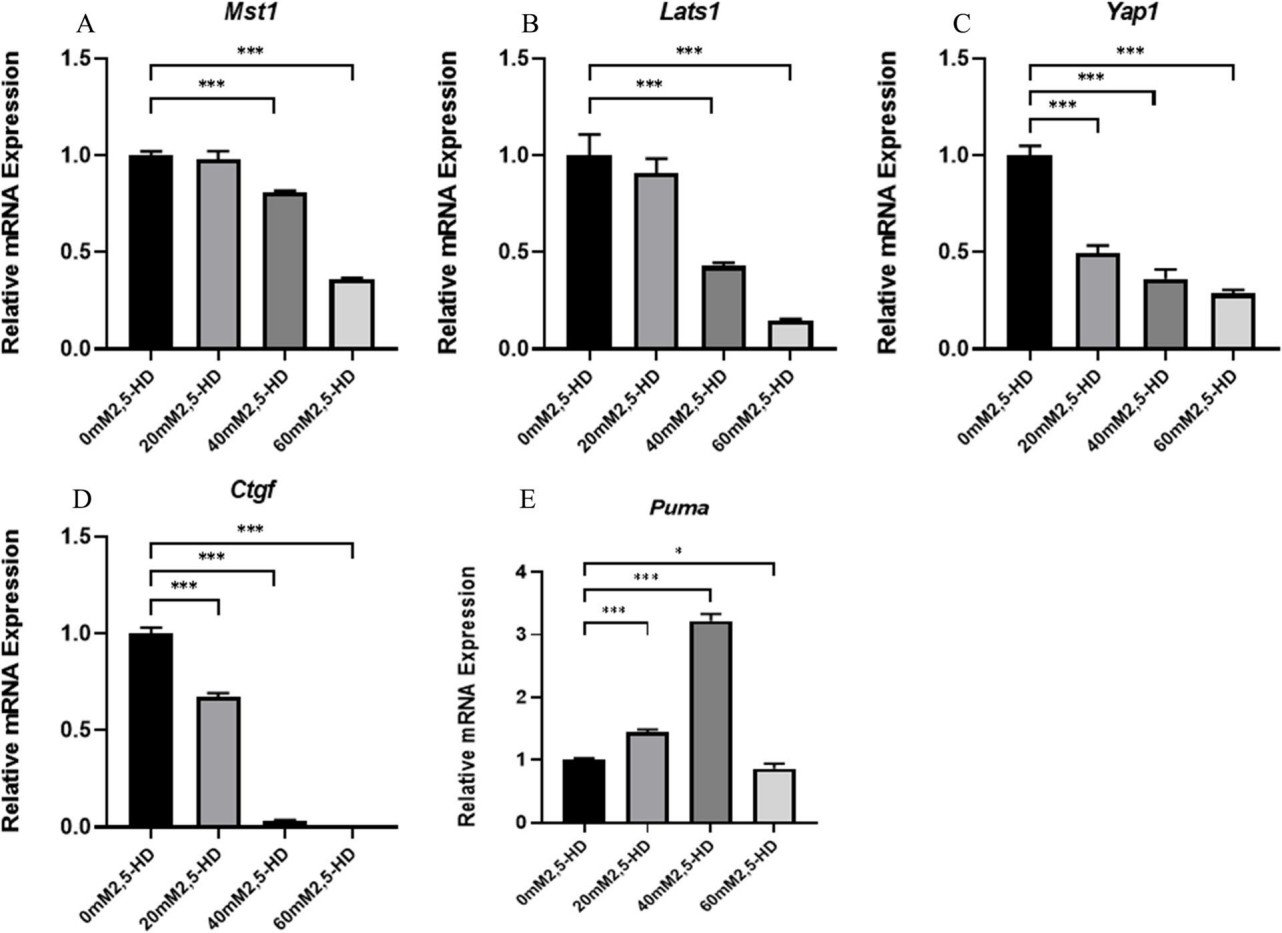
mRNA expression levels of major genes of the Hippo apoptosis-related pathway after 2,5-HD treatment

**Fig. 6:**
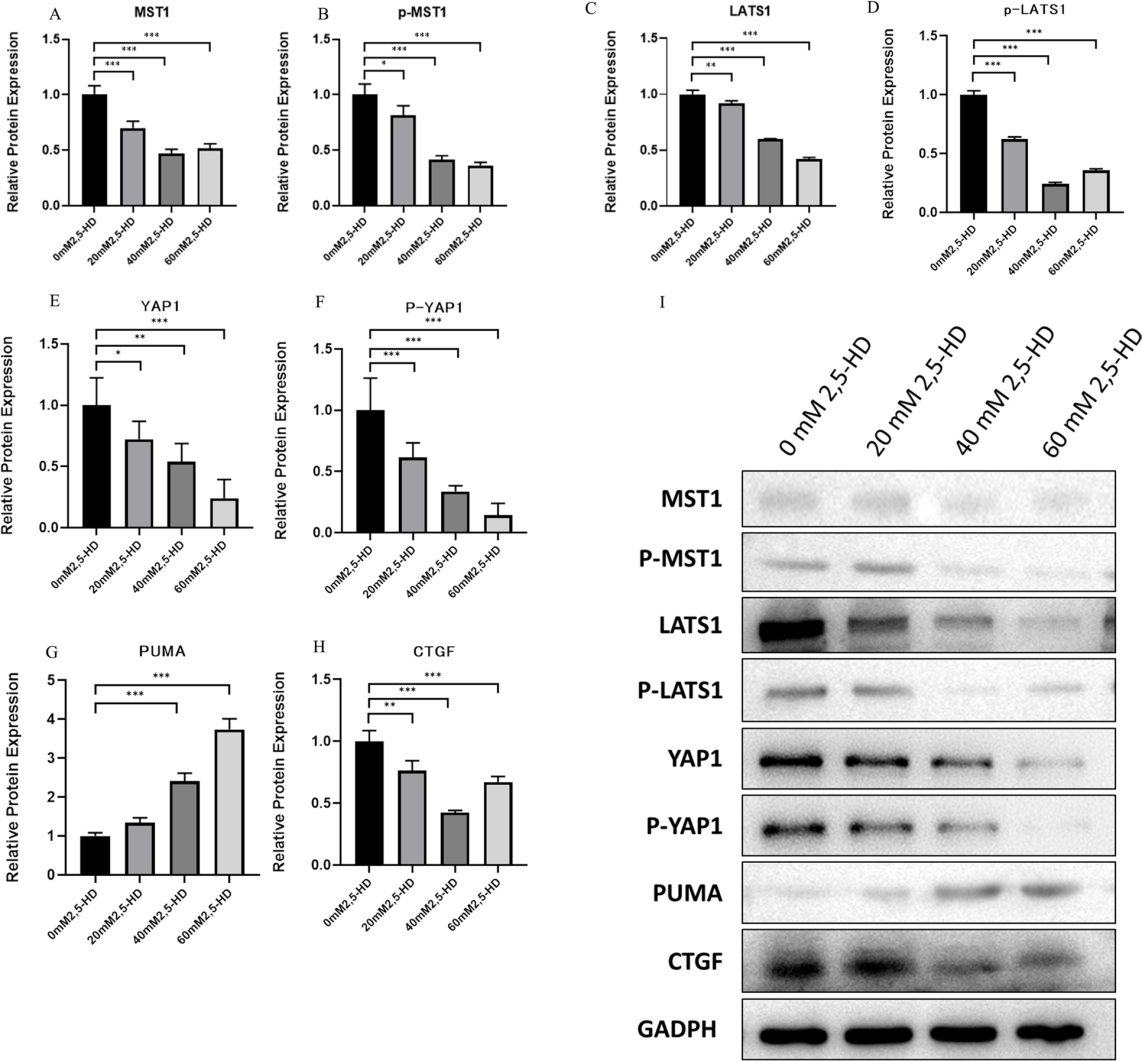
Protein expression levels of major genes of the Hippo apoptosis-related pathway after 2,5-HD treatment

Furthermore, we measured the expression levels of Mst1, Lats1, Yap1, PUMA and Ctgf, which are the main genes in the Hippo signaling pathway, and the qRT-PCR results suggested that compared with the control group, the mRNA relative expression levels of Yap1 and Ctgf decreased with increasing treatment dose (*P* < 0.05). The relative mRNA expression levels of Mst1 and Lats1 in the 40 mM and 60 mM treatment groups decreased (*P* < 0.05), and Puma was upregulated in the 20 mM and 40 mM groups but decreased in the 60 mM group (Fig. 5). Western blotting results suggested that MST1, p-MST1, LATS1, p-LATS1, p-LATS1, YAP1, p-YAP1 and CTGF protein expression decreased in each treatment group compared with the control group (*P* < 0.05); PUMA protein expression was upregulated in the 40 mM and 60 mM treatment groups (*P* < 0.05) (Fig. 6).

### Co-IP verifies the interaction of YAP1 with the transcription factors P73 and TEAD

**Fig. 7:**
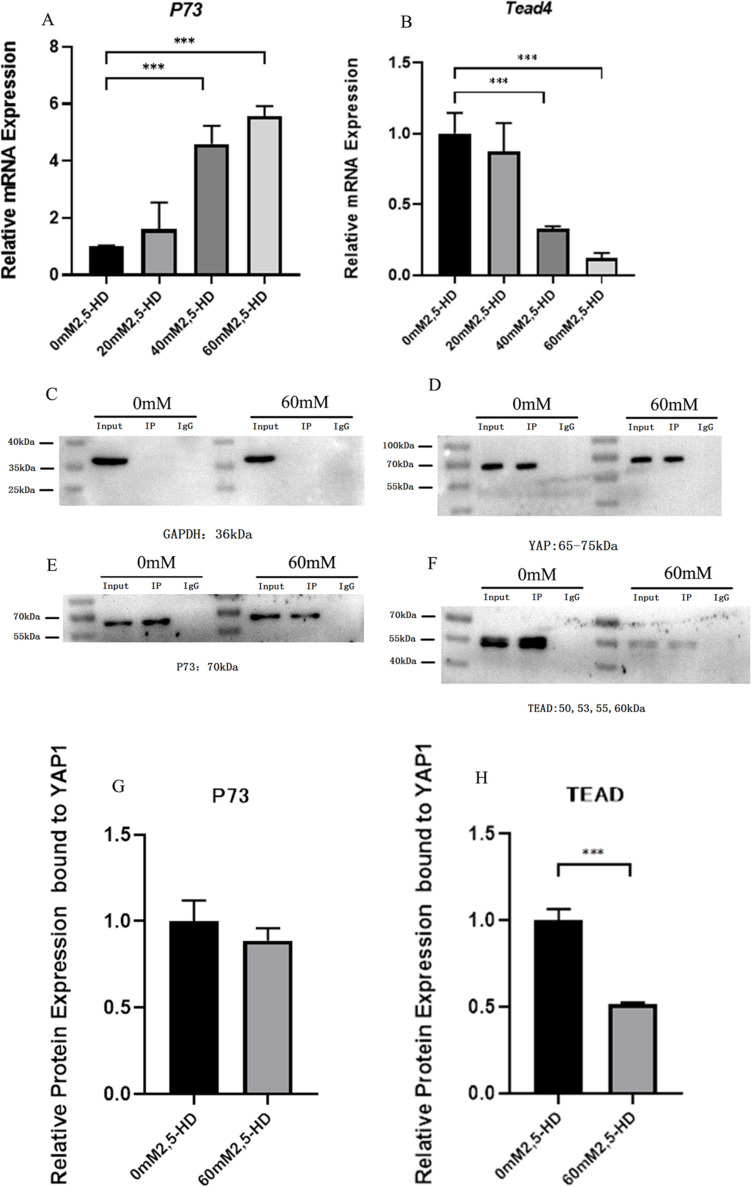
Co-IP verified the interaction of YAP1 with the transcription factors P73 and TEAD. **A**, **B **The mRNA levels of P73 and TEAD4; **C**-**H **CO-IP confirmed the interaction among P73, TEAD and YAP1

Finally, we conducted Co-IP experiments to elucidate the effect of YAP1 on the downstream apoptosis-related intranuclear transcription factors P73 and TEAD, and the results are shown in Fig. 7A, B. The mRNA level of P73 was upregulated in the 40 mM and 60 mM groups, while Tead4 was downregulated in each 2,5-HD treatment group. As shown in Fig. 7C, P73 can be immunoprecipitated with the YAP1 protein, indicating that there is an interaction between P73 and YAP1, but there was no significant difference in the relative amount of the protein that was bound by the transcription factor and YAP1 after 2,5-HD treatment (*P* >0.05). TEAD also interacts with YAP1, but after treatment with 2,5-HD, TEAD protein expression levels were reduced, and the relative amounts of proteins that bound to YAP1 were reduced (*P* <0.05) (Fig. 7C-H).

## Discussion

In this study, we observed by mRNA expression profiling that in ovarian GCs, the Hippo signaling pathway was significantly inhibited during 2,5-HD-induced apoptosis, as shown by the downregulation of pro-proliferation gene expression and the upregulation of pro-apoptosis genes expression. Further mechanistic studies showed that Puma was significantly upregulated after YAP1 entered the nucleus following treatment with 2,5-HD (enhanced proapoptotic signal), but this was not explained by an interaction between YAP1 and P73. In addition, 2,5-HD exposure reduced the interaction between YAP1 and TEAD, thereby inhibiting proliferation signals. The balance between cell proliferation and apoptosis was disrupted, which increased the apoptosis of ovarian GCs.

### Effect of in vitro 2,5-HD exposure on the apoptosis of ovarian GCs

In this study, Hoechst 33258 staining was used to confirm the apoptosis-inducing effect of 2,5-HD on GCs, and the rate of GC apoptosis after treatment with 2,5-HD was higher than in the control group. Flow cytometry is characterized by high sensitivity and rapidity and is an important method for studying apoptosis [19]. Flow cytometry results also showed that the rate of GC apoptosis increased in 2,5-HD treatment group. Thus, 2,5-HD increased the apoptosis of ovarian GCs, which may be one of the mechanisms underlying ovarian function damage.

### mRNA microarray analysis of ovarian GCs in rats treated with 2,5-HD

To investigate the key pathways that regulated the apoptosis induced by 2,5-HD in rat ovarian GCs, we performed mRNA microarray analysis. The results showed that there were 5677 differentially expressed mRNAs between the treated and control groups, and further GO and KEGG analyses suggested that the differentially expressed genes were significantly enriched in Ras signaling pathway, PI3K-Akt signaling pathway, and Hippo signaling pathway. Among these pathways, the Hippo signaling pathway has attracted great interest.

The Hippo signaling pathway plays an important role in regulating cell proliferation and apoptosis [13, 35]. The Hippo signaling pathway is mainly composed of three interrelated parts: upstream regulatory signaling molecules (Fat, FRMD6, NF2, KIBRA, etc.), core kinase cascade complexes (Mst1/2, Lats1/2, TAZ, and YAP), and downstream intranuclear transcriptional regulatory complexes (TEAD1/4, Wbp-2, p73, etc.) [12]. Further, we screened seven genes with significant expression differences in expression that were related to the Hippo pathway, namely, Nf2, Wwc1, Ajuba, Llgl1, Dlg3, Rassf6, and Rassf1. qRT‒PCR showed that these genes were significantly differentially expressed, again suggesting that the Hippo signaling pathway is important in 2,5-HD-induced ovarian GC apoptosis. In addition, we noticed that the qRT-PCR results were basically consistent with the microarray results, which also suggested that the microarray data were reliable.

Nf2, Wwc1, Ajuba, Llgl1, Dlg3, Rassf6 and Rassf1 are upstream signaling molecules of the Hippo signaling pathway. Increased expression of NF2 promotes the activation of MST1 [23], and loss of WWC1 expression can be accompanied by a decrease in LATS and YAP phosphorylation [25]. RASSF6 is a member of the RASSF tumor suppressor protein family that binds to MST1/2 and synergizes with the Hippo pathway to induce apoptosis [3, 34]. RASSF1A increases MST1/2 phosphorylation and promotes MST kinase activity [9]. In addition, downregulation of AJUBA expression is associated with structural activation of YAP, which negatively regulates YAP activity via LATS [32]. Lgl, Scribble, and Dlg from protein complexes, and Scrib/Dlg/Lgl complexes can serve as scaffolds to promote interactions between the proteins of the Hippo pathway [26]. The results showed that the expression of Nf2, Wwc1, Ajuba, Llgl1, Dlg3, Rassf6 and Rassf1 was significantly altered after 2,5-HD treatment, suggesting that changes in the Hippo signaling pathway are likely to be involved in GC apoptosis induced by 2,5-HD exposure.

### Changes in upstream signaling molecules cause changes in the expression of key genes that are related to the Hippo signaling pathway

Next, we wanted to further explore the changes that occurred in the expression of core genes of the Hippo apoptotic pathway, namely, Mst1, Lats1, Yap1, Puma, and Ctgf when Hippo signaling pathway was inhibited by changes in upstream signaling molecules, such as Nf2, Wwc1, Ajuba, Llgl1, Dlg3, Rassf6, and Rassf1.

The results suggested that MST1, LATS1, p-LATS1, and p-MST1 were decreased after 2,5-HD treatment. Combined with the previous findings, the changes in MST1 and LATS1 expression may be due to coregulation by upstream regulatory genes, such as Nf2, Rassf1, and Wwc1, which inhibit MST1 and LATS1 total protein expression as well as their phosphorylation, thereby inhibiting the Hippo signaling pathway [16, 27, 37]. Numerous studies have suggested that MST1 and LATS1 are associated with changes in YAP [33]. For example, it has been shown that overexpression of LATS1 in ovarian GCs reduces estrogen secretion, and LATS1 can regulate StAR expression by phosphorylating LOXL2 and YAP [5]. Our results showed that the protein expression of YAP1 and p-YAP1 was decreased in each treatment group, indicating that YAP1 was related to 2,5-HD-induced apoptosis in rat ovarian GCs. This may be associated with downregulation of MST1 and LATS1.

Subsequently, we measured the expression levels of the downstream target proteins CTGF and PUMA after the translocation of activated YAP to the nucleus during 2,5-HD-mediated inhibition of the Hippo signaling pathway, and the results showed that the expression of the pro-proliferation protein CTGF was decreased and the expression of the pro-apoptosis protein PUMA was increased, which were closely related to 2,5-HD-induced ovarian GC apoptosis. How does YAP cause changes in the expression of downstream target genes after entering the nucleus?

### Molecular mechanism underlying the interaction between major proteins of the Hippo apoptotic pathway during 2,5-HD-induced apoptosis in rat ovarian GCs

It has been shown that p73-induced apoptosis is mediated by PUMA, which in turn leads to the mitochondrial translocation of Bax, and p73 induces apoptosis through the mitochondrial pathway using PUMA and BAX as mediators [4, 7, 24]. After entering the nucleus, YAP can bind to P73 and promote the transcription of the apoptotic gene Puma [4]. In addition, When the Hippo pathway is inhibited, YAP, when bound to the transcription factor TEAD, can promote the expression of the downstream target gene Ctgf expression and participate in cell proliferation and apoptosis [38, 40, 42]. This suggests that the changes in Ctgf and Puma expression are likely related to the effects of YAP1 interaction with P73 and TEAD.

Therefore, we further investigated whether YAP1 interacts with the P73 and TEAD proteins. First, we found that 2,5-HD treatment also affected the expression of p73 and Tead. Subsequent Co-IP results showed that P73 could coimmunoprecipitate with the YAP1 protein in this experimental treatment model, indicating that P73 interacted with YAP1, however, 2,5-HD treatment did not affect the binding of YAP1 to the apoptosis-related transcription factor P73. In addition, YAP1 can also interact with TEAD, and after treatment 2,5-HD, the interaction of YAP1 with TEAD was significantly reduced, which may result in weakened downstream cell proliferation signals. Thus, 2,5-HD exposure promoted the nuclear translocation of activated YAP, which resulted in decreased expression of the downstream target protein CTGF; this may be associated with decreased interactions between YAP and TEAD, while expression of the proapoptotic gene PUMA increased, regardless of the direct interaction of YAP with P73.

In summary, inhibition of the Hippo signaling pathway is critical during the 2,5-HD-induced apoptosis of ovarian GCs under the conditions of this experiment. The expression levels of MST1 and LATS1 were reduced, and the inhibitory effect on YAP1 was lost, causing a decrease in the downstream target protein pro-proliferation CTGF protein and an increase in the expression level of pro-apoptotic PUMA. The decreased interaction between YAP1 to the TEAD transcription factor is responsible for the decreased expression of the anti-apoptotic protein CTGF. However, upregulation of PUMA was not associated with the direct binding of YAP to P73. The Hippo signaling pathway is likely to be involved in the 2,5-HD-induced apoptosis of rat ovarian GCs by regulating the balance between cell proliferation and apoptosis. However, due to the lack of experiments in which the Hippo signaling pathway was targeted, it is not known whether 2,5-HD-induced GC apoptosis would change after activation of the Hippo pathway. For example, it is necessary to carry out further YAP1 rescue experiments to further elucidate the mutual regulatory relationship between genes.

### Supplementary Information

**Additional file 1: Table S1.** Primer sequences.

**Additional file 2: Figure S1.** Changes in cell viability after 24 h of treatment with 2,5-HD.

**Additional file 3: Figure S2.** mRNA microarray analysis. A: Scatter plot, B: Volcano plot, C: Cluster plot (0 mM group: C1, C3, C4; 60 mM group: A1, A2, A5). In the figure, green dots indicate differentially expressed genes with downregulated expression after 2,5-HD exposure, red dots indicate differentially expressed genes with upregulated expression, and black dots indicate genes with no significant difference in expression.

## Data Availability

The relevant data and Additional files 1, 2 and 3 are all availed.

## References

[1] Abolaji AO, Adedara IA, Soladogun A, Salau V, Oguaka M, Farombi EO (2015). Exposure to 2,5-hexanedione is accompanied by ovarian and uterine oxidative stress and disruption of endocrine balance in rats. Drug Chem Toxicol.

[2] Abouhashem N, Harb O, Elwan A, Zaitoun M, Saraya Y (2022). Immunohistochemical evaluation of forkhead box A1 and EphA5 markers in serous ovarian carcinomas, and their impact on the clinical outcome of patients. Pol J Pathol.

[3] Allen NP, Donninger H, Vos MD, Eckfeld K, Hesson L, Gordon L, Birrer MJ, Latif F, Clark GJ (2007). RASSF6 is a novel member of the RASSF family of tumor suppressors. Oncogene.

[4] Basu S, Totty NF, Irwin MS, Sudol M, Downward J (2003). Akt phosphorylates the Yes-associated protein, YAP, to induce interaction with 14-3-3 and attenuation of p73-mediated apoptosis. Mol Cell.

[5] Cai JH, Sun YT, Bao S (2022). HucMSCs-exosomes containing miR-21 promoted estrogen production in ovarian granulosa cells via LATS1-mediated phosphorylation of LOXL2 and YAP. Gen Comp Endocrinol.

[6] Clark KL, George JW, Hua G, Davis JS (2022). Perfluorooctanoic acid promotes proliferation of the human granulosa cell line HGrC1 and alters expression of cell cycle genes and Hippo pathway effector YAP1. Reprod Toxicol.

[7] Deng LJ, Qi M, Peng QL, Chen MF, Qi Q, Zhang JY, Yao N, Huang MH, Li XB, Peng YH, Liu JS, Fu DR, Chen JX, Ye WC, Zhang DM (2018). Arenobufagin induces MCF-7 cell apoptosis by promoting JNK-mediated multisite phosphorylation of Yes-associated protein. Cancer Cell Int.

[8] Fu D, Lv X, Hua G, He C, Dong J, Lele SM, Li DW, Zhai Q, Davis JS, Wang C (2014). YAP regulates cell proliferation, migration, and steroidogenesis in adult granulosa cell tumors. Endocr Relat Cancer.

[9] Guo C, Zhang X, Pfeifer GP (2011). The tumor suppressor RASSF1A prevents dephosphorylation of the mammalian STE20-like kinases MST1 and MST2. J Biol Chem.

[10] Hao EY, Wang DH, Chang LY, Huang CX, Chen H, Yue QX, Zhou RY, Huang RL (2020). Melatonin regulates chicken granulosa cell proliferation and apoptosis by activating the mTOR signaling pathway via its receptors. Poult Sci.

[11] Heijink E, Scholten SW, Bolhuis PA, de Wolff FA (2000). Effects of 2,5-hexanedione on calpain-mediated degradation of human neurofilaments in vitro. Chem Biol Interact.

[12] Hilman D, Gat U (2011). The evolutionary history of YAP and the hippo/YAP pathway. Mol Biol Evol.

[13] Hong AW, Meng Z, Guan KL (2016). The Hippo pathway in intestinal regeneration and disease. Nat Rev Gastroenterol Hepatol.

[14] Hu LL, Su T, Luo RC, Zheng YH, Huang J, Zhong ZS, Nie J, Zheng LP (2019). Hippo pathway functions as a downstream effector of AKT signaling to regulate the activation of primordial follicles in mice. J Cell Physiol.

[15] Huang J, Wu S, Barrera J, Matthews K, Pan D (2005). The Hippo signaling pathway coordinately regulates cell proliferation and apoptosis by inactivating Yorkie, the Drosophila Homolog of YAP. Cell.

[16] Kim SH, Jin H, Meng RY, Kim DY, Liu YC, Chai OH, Park BH, Kim SM (2019). Activating hippo pathway via Rassf1 by ursolic acid suppresses the tumorigenesis of gastric cancer. Int J Mol Sci.

[17] Li X, Yu T, Wang S, Wang Q, Li M, Liu Z, Xie K (2020). Diallyl sulfide-induced attenuation of n-hexane-induced peripheral nerve impairment is associated with metabolic inhibition of n-hexane. Food Chem Toxicol.

[18] Liu J, Huang HL, Pang F, Zhang WC (2012). The effect of n-hexane on the gonad toxicity of female mice. Biomed Environ Sci.

[19] Liu J, Liao J, Zhang C, Zeng L, Zong C, Lv Y, Li J, Zhang W (2021). The role of miRNAs in regulating the effect of prenatal cadmium exposure on ovarian granulosa cells in a transgenerational manner in female rats. Food Chem Toxicol.

[20] Liu J, Zhang W (2019). Methods for evaluation of ovarian granulosa cells with exposure to nanoparticles. Methods Mol Biol.

[21] LoPachin RM, DeCaprio AP (2004). gamma-Diketone neuropathy: axon atrophy and the role of cytoskeletal protein adduction. Toxicol Appl Pharmacol.

[22] Markström E, Svensson ECH, Shao R, Svanberg B, Billig H (2002). Survival factors regulating ovarian apoptosis – dependence on follicle differentiation. Reproduction.

[23] Matsuda T, Zhai P, Sciarretta S, Zhang Y, Jeong JI, Ikeda S, Park J, Hsu CP, Tian B, Pan D, Sadoshima J, Del Re DP (2016). NF2 activates hippo signaling and promotes ischemia/reperfusion injury in the heart. Circ Res.

[24] Melino G, Bernassola F, Ranalli M, Yee K, Zong WX, Corazzari M, Knight RA, Green DR, Thompson C, Vousden KH (2004). p73 Induces apoptosis via PUMA transactivation and bax mitochondrial translocation. J Biol Chem.

[25] Moleirinho S, Chang N, Sims AH, Tilston-Lünel AM, Angus L, Steele A, Boswell V, Barnett SC, Ormandy C, Faratian D, Gunn-Moore FJ, Reynolds PA (2013). KIBRA exhibits MST-independent functional regulation of the Hippo signaling pathway in mammals. Oncogene.

[26] Penzo-Méndez AI, Stanger BZ (2014). Cell competition in vertebrate organ size regulation. Wiley Interdiscip Rev Dev Biol.

[27] Qi S, Zhu Y, Liu X, Li P, Wang Y, Zeng Y, Yu A, Wang Y, Sha Z, Zhong Z, Zhu R, Yuan H, Ye D, Huang S, Ling C, Xu Y, Zhou D, Zhang L, Yu FX (2022). WWC proteins mediate LATS1/2 activation by Hippo kinases and imply a tumor suppression strategy. Mol Cell.

[28] Ruiz-García L, Figueroa-Vega N, Malacara JM, Barrón-Vivanco B, Salamon F, Carrieri M, Jiménez-Garza O (2020). Possible role of n-hexane as an endocrine disruptor in occupationally exposed women at reproductive age. Toxicol Lett.

[29] Sun LC, Xia LJ, Meng XP, Liu L, Gao XH, Yang GC (2006). Protective effect of mouse 2.5s nerve growth factor on PC12 cells from injury induced by 2, 5-hexanedione. Zhonghua Lao Dong Wei Sheng Zhi Ye Bing Za Zhi.

[30] Sun Y, Lin Y, Li H, Liu J, Sheng X, Zhang W (2012). 2,5-Hexanedione induces human ovarian granulosa cell apoptosis through BCL-2, BAX, and CASPASE-3 signaling pathways. Arch Toxicol.

[31] Sun Y, Lv Y, Li Y, Li J, Liu J, Luo L, Zhang C, Zhang W (2022). Maternal genetic effect on apoptosis of ovarian granulosa cells induced by cadmium. Food Chem Toxicol.

[32] Tanaka I, Osada H, Fujii M, Fukatsu A, Hida T, Horio Y, Kondo Y, Sato A, Hasegawa Y, Tsujimura T, Sekido Y (2015). LIM-domain protein AJUBA suppresses malignant mesothelioma cell proliferation via Hippo signaling cascade. Oncogene.

[33] Tsoi M, Morin M, Rico C, Johnson RL, Paquet M, Gévry N, Boerboom D (2019). Lats1 and Lats2 are required for ovarian granulosa cell fate maintenance. FASEB J.

[34] Withanage K, Nakagawa K, Ikeda M, Kurihara H, Kudo T, Yang Z, Sakane A, Sasaki T, Hata Y (2012). Expression of RASSF6 in kidney and the implication of RASSF6 and the Hippo pathway in the sorbitol-induced apoptosis in renal proximal tubular epithelial cells. J Biochem.

[35] Xiang C, Li J, Hu L, Huang J, Luo T, Zhong Z, Zheng Y, Zheng L (2015). Hippo signaling pathway reveals a spatio-temporal correlation with the size of primordial follicle pool in mice. Cell Physiol Biochem.

[36] Xue LX, Chen SF, Xue SX, Zhang XZ, Lian YJ (2022). P2RY2 alleviates cerebral ischemia-reperfusion injury by inhibiting YAP phosphorylation and reducing mitochondrial fission. Neuroscience.

[37] Yang W, Han W, Qin A, Wang Z, Xu J, Qian Y (2018). The emerging role of Hippo signaling pathway in regulating osteoclast formation. J Cell Physiol.

[38] Ye XY, Luo QQ, Xu YH, Tang NW, Niu XM, Li ZM, Shen SP, Lu S, Chen ZW (2015). 17-AAG suppresses growth and invasion of lung adenocarcinoma cells via regulation of the LATS1/YAP pathway. J Cell Mol Med.

[39] Zilz TR, Griffiths HR, Coleman MD (2007). Apoptotic and necrotic effects of hexanedione derivatives on the human neuroblastoma line SK-N-SH. Toxicology.

[40] Zhang J, Ji JY, Yu M, Overholtzer M, Smolen GA, Wang R, Brugge JS, Dyson NJ, Haber DA (2009). YAP-dependent induction of amphiregulin identifies a non-cell-autonomous component of the Hippo pathway. Nat Cell Biol.

[41] Zhang W, Huang L, Kong C, Liu J, Luo L, Huang H (2013). Apoptosis of rat ovarian granulosa cells by 2,5-hexanedione in vitro and its relevant gene expression. J Appl Toxicol.

[42] Zhu C, Li L, Zhang Z, Bi M, Wang H, Su W, Hernandez K, Liu P, Chen J, Chen M, Huang TH, Chen L, Liu Z (2019). A non-canonical role of YAP/TEAD is required for Aactivation of estrogen-regulated enhancers in breast cancer. Mol Cell.

