# Supplementary material for: The Hippo signaling pathway contributes to the 2,5-Hexadion-induced apoptosis of ovarian granulosa cells
Source: J Ovarian Res. 2023 Aug 11;16:161. doi: 10.1186/s13048-023-01249-4 (PMC10416496; doi:10.1186/s13048-023-01249-4)
Supplement: Supplementary file 1 — Additional file 1: Table S1. Primer sequences. [file 13048_2023_1249_MOESM1_ESM.docx]

Table S1 Primer Sequences

| Gene sympol | Primer sequence（5' to 3'） | |
| --- | --- | --- |
| *Gadph* | Forward | 5'- GTTACCAGGGCTGCCTTCTC-3' |
|  | Reverse | 5'- GATGGTGATGGGTTTCCCGT-3' |
| *Nf2* | Forward | 5'- CATCAAAAAGCCTCAAGCCCA-3' |
|  | Reverse | 5'- TGGTCGGGTCACCTAGAGTT-3 |
| *Wwc1* | Forward | 5'- CAACCCGCTGGTACAACCTT-3' |
|  | Reverse | 5'- ACGGCATCCACATGGTCGG-3' |
| *Ajuba* | Forward | 5'- CTACCACTGTGAGGACTGCC-3' |
|  | Reverse | 5'- CAGTGGTGATGGCAGCAGTA-3' |
| *Llgl1* | Forward | 5'- ATGCCATCTGGTCCACAGAC-3' |
|  | Reverse | 5'- GTGGTCTCCTGACTCACAGC-3' |
| *Dlg3* | Forward | 5'- CCCAGCCTATCGGTGAATGG-3' |
|  | Reverse | 5'- GATGCCACCTGCGATACTGA-3' |
| *Tead4* | Forward | 5'- GAGTTCCTGCTTCCTGCTCC-3' |
|  | Reverse | 5'- AATGGGCGCTCCAGCTTC-3' |
| *Rassf6* | Forward | 5'- TTAGCAGTGATGTGGCTCCG-3' |
|  | Reverse | 5'- CCGTTTTTGCTGCCCGTTTA-3' |
| *Rassf1* | Forward | 5'- CTGGGGAGGTGAATTGGGATG-3' |
|  | Reverse | 5'- TCTGGCGACACCGAGAATAC-3' |
| *Puma* | Forward | 5'- CAACTAGGTGCCTACACCCG-3' |
|  | Reverse | 5'- CAAGGCTGGCAGTCCAGTAT-3' |
| *Yap1* | Forward | 5'- AATGTCAGACCGTCAGAGCG-3' |
|  | Reverse | 5'- GTCATCCCGGGAGAAGACAC-3 |
| *Mst1* | Forward | 5'- ATGACCACAAGTACACGCCC-3' |
|  | Reverse | 5'- CTACCTCGCCACGGTAATCC-3' |
| *Lats1* | Forward | 5'- CTACGGACAGGATATACACAGC-3' |
|  | Reverse | 5'- CTTGGCTTGAGGTGGGATGT-3' |
| *Ctgf* | Forward | 5'- GACAACCCCAGGAGCCAC-3' |
|  | Reverse | 5'- GTGGCAGGCCGGGTG-3' |
| *P73* | Forward | 5'- TGGTGTCAACAAACTGCCCT-3' |
|  | Reverse | 5'- GTATCCCGGAACCCATGGAC-3' |
