# Supplementary figures and images for: The Hippo signaling pathway contributes to the 2,5-Hexadion-induced apoptosis of ovarian granulosa cells
Source: J Ovarian Res. 2023 Aug 11;16:161. doi: 10.1186/s13048-023-01249-4 (PMC10416496; doi:10.1186/s13048-023-01249-4)

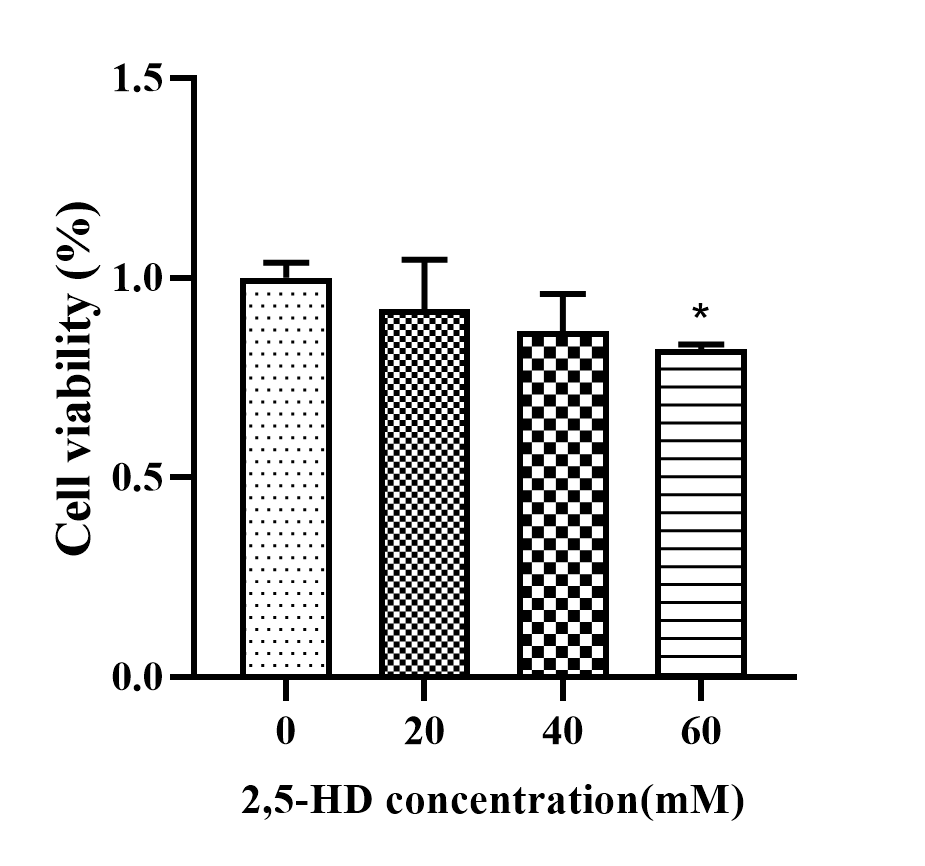


**Figure S1** Changes in cell viability after 24 h of treatment with 2,5-HD.

Supplement: Supplementary file 2 — Additional file 2: Figure S1. Changes in cell viability after 24 h of treatment with 2,5-HD. [file 13048_2023_1249_MOESM2_ESM.docx]
