# Supplementary material for: The Hippo signaling pathway contributes to the 2,5-Hexadion-induced apoptosis of ovarian granulosa cells
Source: J Ovarian Res. 2023 Aug 11;16:161. doi: 10.1186/s13048-023-01249-4 (PMC10416496; doi:10.1186/s13048-023-01249-4)
Supplement: Supplementary file 3 — Additional file 3: Figure S2. mRNA microarray analysis. A: Scatter plot, B: Volcano plot, C: Cluster plot (0 mM group: C1, C3, C4; 60 mM group: A1, A2, A5). In the figure, green dots indicate differentially expressed genes with downregulated expression after 2,5-HD exposure, red dots indicate differentially expressed genes with upregulated expression, and black dots indicate genes with no significant difference in expression. [file 13048_2023_1249_MOESM3_ESM.docx]

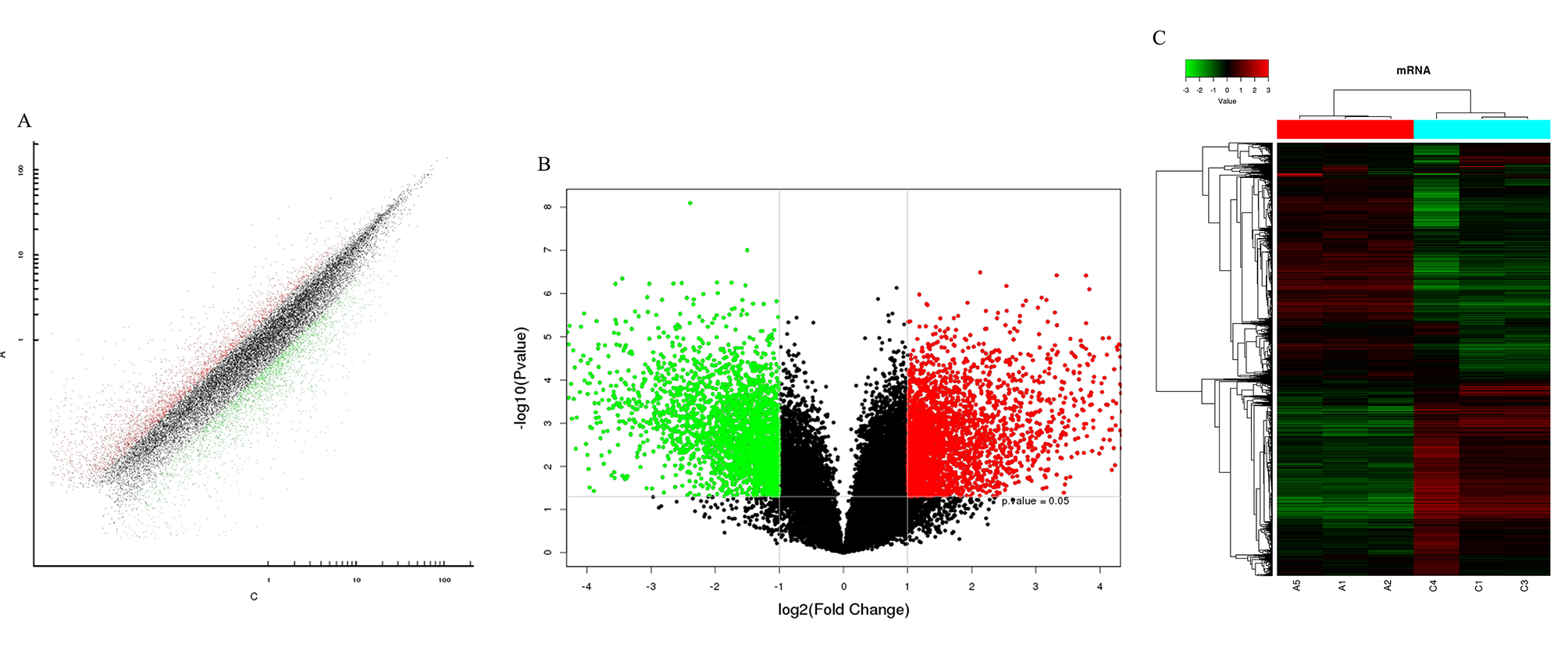


**Figure S2** Figure S2 mRNA microarray analysis. A: Scatter plot, B: Volcano plot, C: Cluster plot (0 mM group: C1, C3, C4; 60 mM group: A1, A2, A5). In the figure, green dots indicate differentially expressed genes with downregulated expression after 2,5-HD exposure, red dots indicate differentially expressed genes with upregulated expression, and black dots indicate genes with no significant difference in expression.
